# Protocol for a scoping review of measurement of sleep in mild cognitive impairment and early dementia

**DOI:** 10.12688/amrcopenres.12981.1

**Published:** 2021-04-14

**Authors:** Jonathan Blackman, Hamish Morrison, Sam Harding, Katherine Lloyd, Elizabeth Coulthard

**Affiliations:** 1 Neurology, North Bristol NHS Trust, Bristol, BS10 5NB, UK; 2 Bristol Medical School, University of Bristol, Bristol, BS2 8DZ, UK; 3 Bristol Speech and Language Therapy Research Unit, North Bristol NHS Trust, Bristol, BS10 5NB, UK

**Keywords:** Sleep, Dementia, Mild Cognitive Impairment, MCI

## Abstract

**Background:**

Sleep abnormalities are increasingly recognised to emerge early in dementia, at or before the Mild Cognitive Impairment (MCI) phase. Abnormal sleep accelerates cognitive decline and may directly contribute to pathophysiology. Its accurate measurement is therefore crucial, firstly to characterise sleep abnormalities in early disease potentially facilitating earlier identification of those at risk of dementia and secondly to test sleep intervention efficacy. However, it is our a priori hypothesis that sleep outcomes are reported heterogeneously inhibiting side-by-side comparison of study findings. As a translational step towards informing choice and decisions on optimal measures, this scoping review will describe measurement tools utilised and sleep parameters currently reported in early dementia and MCI.

**Methods:**

This scoping review follows the Joanna Briggs Institute Manual for Evidence Synthesis for Scoping Reviews. The search strategy consists of an electronic search of the CINAHL Plus, Embase, Medline, Psychinfo and British Nursing Index databases and date limited to articles published from 2000. Search results will be merged using reference management software and duplicates removed. 10% of returned titles and abstracts will be checked by each reviewing member to ensure continuity of decision making. Full-texts will be reviewed by at least two reviewers with discrepancies resolved by whole team consensus. A PRISMA flow diagram will document the selection process. Extracted data will be analysed and reported narratively.

**Discussion:**

This scoping review will identify which sleep parameters are reported and the means by which they are measured in people with MCI or early dementia. We intend to explore differences in reporting practice within group subsets, e.g. by dementia and study subtype.

**Ethics and dissemination:**

Ethical approval is not required due to absence of human participants. Results will be published in a peer-reviewed journal and presented at relevant academic conferences. The search strategy will be made available publicly for transparency.

## Introduction

Sleep abnormalities and circadian rhythm disturbance are well recognised in established dementia ^1– 5^. These include objectively measured micro and macro-architectural disturbances of sleep, alongside subjective reports of reduced quantity and quality ^2^, all of which are disproportionately represented compared with age-matched controls and correlate closely with the severity of cognitive impairment ^6, 7^. Circadian rhythm disorders, contributing to sleep disturbance are also generally more marked in dementia than in healthy ageing – possibly related to volumetric alterations in the Suprachiasmatic Nucleus influencing melatonin secretion ^8^. 

As opposed to solely representing a marker of established disease, sleep abnormalities are increasingly recognised to occur much earlier in the natural history of dementia, during and even preceding the Mild Cognitive Impairment (MCI) stage ^9, 10^. Furthermore, conditions with sleep abnormalities but normal cognition e.g. chronic insomnia, increase future risk of Alzheimer’s Disease (AD) dementia ^11, 12^. In Lewy body dementia, sleep disturbances also precede disease onset. Rapid eye movement-sleep behaviour disorder can present several years before disease manifestation and predict cognitive impairment ^13^.

Whilst such abnormalities in sleep may reflect early symptomatic manifestation of pathology, there are also plausible mechanisms by which sleep abnormalities could precipitate or accelerate pathophysiological decline ^14– 18^. In AD, sleep abnormalities have been hypothesised to contribute to diminished clearance of a key pathognomonic feature - beta-amyloid ^17, 18^, supported by work showing the unique role of Slow Wave Sleep (SWS) in removing intracerebral toxic breakdown products (including beta amyloid) in mice ^19^. Supporting this, SWS disruption in both healthy adults and those with AD is associated with greater levels of beta-amyloid pathology ^20^.

The pathological changes associated with multiple subtypes of dementia predate symptomatic expression of symptoms by decades ^21, 22^ and as such a promising future strategy will be targeting early stages of the disease when pathology is more likely to be reversible and quality of life can be retained. Given that sleep abnormalities arise early, they may provide an ideal means to identify those at highest risk of dementia and this early identification is potentially crucial when implementing therapeutic modalities to alter the pathological disease course in the future. In addition, optimising sleep itself is hypothesised as an opportunity to delay progression of neurodegenerative disease whilst simultaneously promoting physiological processes that improve cognition (particularly long-term memory consolidation), general health and wellbeing. As a result, there is much current interest in enhancing understanding of the precise nature of sleep abnormalities in dementia, their presence prior to onset of clinical symptoms and trials of interventions to improve sleep disturbances.

However, whilst providing a rich vein of opportunity for deeper characterisation and intervention, sleep is a challenging concept to measure in dementia - both due to its complex nature and the target population. As a multidimensional concept, sleep is measurable across levels and aspects ^23, 24^. For example, levels of measurement may include self-report questionnaires, behavioural measures, e.g. actigraphy, physiological means, e.g. polysomnography, and less commonly analyses at the circuit or cellular level. For the purposes of this review these will be referred to as measurement tools. Within each level, multiple aspects may be recorded e.g. sleep duration, efficiency etc., which for the purpose of this review will be referred to as sleep parameters. Furthermore, measuring sleep in MCI and early dementia is unique as it encompasses challenges not seen in healthy populations ^25^, whilst also allowing for a wider range of techniques when compared to those in later stage disease. 

It is our a priori hypothesis that these factors ally to potentially compromise comparison and reproducibility of work designed to facilitate detailed characterisation of sleep and also to assess the effects of interventions. A recent systematic review reporting objective sleep measurement findings in MCI was unable to render specific conclusions relating to micro-architectural sleep as no two studies were found to report the same parameters ^10^. Similarly a systematic review exploring sleep interventions in MCI was confined to narrative review due to outcome measure heterogeneity ^26^.

There is a pressing need to develop a recommended outcome set of sleep parameters and the optimal means by which they can be measured. As a translational step towards this, a scoping review was considered the most appropriate framework in exploring current research conduct within the field. To our knowledge, to date, no reviews exist describing current practices in measuring and reporting sleep within this population in clinical settings, or in interventional and observational studies, supported by an absence of systematic reviews or scoping reviews found through preliminary searches of MEDLINE, EMBASE and PROSPERO. 

The primary objective of this scoping review therefore aims to address this gap in the literature by exploring which sleep outcome variables are reported and the means by which they are measured in the current literature. Secondarily it aims to describe how this differs in interventional vs. observational studies and amongst separate categories/types of MCI/early dementia. 

## Protocol

### Review question

“How is sleep currently measured and reported in the literature from studies involving participants with Mild Cognitive Impairment (MCI) and early dementia?”

### Eligibility criteria

***Participants***

Inclusion criteria:

1. Adults aged greater than 18 (limit set to avoid excluding studies in genetic dementias); and
2. Male or Female; and
3. a) Satisfies established diagnostic criteria for MCI or minor neurocognitive disorder e.g. Albert Criteria,Peterson Criteria, DSM V.
      b) Satisfies established diagnostic criteria for dementia or major neurocognitive disorder e.g. DSM-IV, ICD-10, ICD-11, NINCDSARDA, DSM-V.
4. The majority (≥50%) of the study group has mild/early dementia/MCI as evidenced by:
  a) MMSE ≥ 20
  b) CDR < 2
  c) Equivalent measure

***Concept***

All included studies will meet the following two criteria:

1. Sleep measurement/assessment is a key component of interest as evidenced by one or more objective relating to sleep defined within the original aims and objectives of the study.
AND
2. Sleep outcomes/parameters e.g. total sleep time, sleep efficiency, subjective experience of sleep are reported through use of a sleep outcome measure/tools which demonstrate efficacy and have been validated.

***Context.*** This review aims to capture studies conducted in community and health-care settings.

***Types of evidence sources.*** This scoping review will consider published, peer-reviewed articles written in English. Specifically, those reporting both experimental and quasi-experimental study designs including randomised controlled trials, before and after studies and interrupted time-series studies. In addition, analytical observational studies including prospective and retrospective cohort studies, case-control studies and analytical cross-sectional studies as well as descriptive observational study designs including case series, individual case reports and descriptive cross-sectional studies will be considered for inclusion alongside qualitative studies. Review papers, text and opinion papers are ineligible. 

This proposed scoping review will be conducted in accordance with the Joanna Briggs Institute (JBI) methodology for scoping reviews ^27^. 

### Search strategy

An initial search of MEDLINE and EMBASE via Ovid was undertaken to identify relevant articles utilising an initial search strategy (see Box 1). The text words contained in titles and abstracts of a selection of relevant articles will be used to inform the full search strategy. The search strategy, including all identified keywords and index terms will be adapted for each included database.

**Box 1. Preliminary search strategy**

1.   (Sleep* or slept or insomni*).ab. or (Sleep* or slept or insomni*).ti.

2.   Sleep/ or exp insomnia/

3.   1 or 2

4.   (MCI or MNCD or AAMI or AACD or MCD or ARCD or NMCI or AMCI or MMCI or SMCI or MCIA or LBD or FTLD or FTD).ti,ab.

5.   exp Dementia/

6.   (dement* or alzheim* or lewy or frontotemporal).ti,ab.

7.   ((Cognit* or Neurocognit* or Neurodegenerat*) adj3 (disorder* or impair* or declin* or dement* or deficit* or dysfunction* or disease* or impair*)).ti,ab.

8.   4 or 5 or 6 or 7

9.   3 and 8

10.   exp animals/ not exp humans/

11.   9 not 10

12.   11

13.   limit 12 to english language

14.   (measur* or assess* or question* or evaluat* or quanti* or apprais* or guage* or guaging or test* or examin* or inventor* or survey*).ti,ab.

15.   9 and 14

16.   15 not 10

17.   16

18.   limit 17 to english language

19.   limit 18 to articles published after 1999

Due to the substantial number of included articles anticipated, reference searching within included articles will not be performed. In order to ensure that this review reflects contemporaneous practice, the search will be limited to articles published after 1999, at which point objective measurement of sleep became more routinely available. 

An electronic search of the CINAHL Plus, Embase, Medline, PsychInfo and British Nursing Index databases will be performed.

### Selection of eligible studies

Following the search, all identified citations will be collated and uploaded into reference management software with duplicates removed. Titles of studies clearly unrelated to the participants and concept of the scoping review will also be removed. Two reviewers will independently review 10% of the remaining abstracts against the inclusion criteria as stated. They will meet to compare their selection of articles. If agreement is above 90% between the two reviewers for at least 10% of the papers, one reviewer will review the remaining abstracts. If agreement does not reach that level, then a further 10% will be reviewed by the two reviewers and further discussion held. This process will be repeated until there is less than 10% disagreement. Once all abstracts have been reviewed, potentially relevant sources for full text review will then be retrieved in full and their citation details imported into JBI system for the Unified Management, Assessment and Review of Information (SUMARI) ^28^. The two reviewers will review all papers independently at full text level with regular consensus meetings. Reasons for the exclusion of sources at full text will be recorded and reported in the scoping review. Any disagreements that arise between the reviewers at each stage of the selection process will be resolved through either discussion or with an additional reviewer/s. The selection process will be reported in full within the final scoping review and presented in a Preferred Reporting Items for Systematic Reviews and Meta-Analyses extension for scoping review (PRISMA-ScR) flow diagram ^29^. 

### Data extraction

Data will be extracted from papers included in the scoping review by a member of the reviewing team utilising a data extraction tool developed by the reviewers (see Table 1). The data extracted will include details of the participants, study methods, concept, context and key findings regarding outcome measures pertinent to the review question. Any modifications required during the process of data extraction will be detailed within the full scoping review. Attempts will be made to contact authors to resolve issues relating to missing, unclear or incomplete data. This review is designed to highlight sleep outcomes reported in the literature rather than evaluate study quality, as such critical appraisal and risk of bias analysis will not be undertaken. 

**Table 1.** Data extraction form. Adapted from ^27^.

Scoping review details					
Scoping Review Title	Measurement of Sleep in Mild Cognitive Impairment and Early Dementia: A Scoping Review					
Review Question	“How is sleep currently measured and reported in studies involving participants with Mild Cognitive Impairment (MCI) and early dementia?”					
Inclusion/exclusion criteria					
Population	1. Adults > 18 2. Male or Female 3. Satisfies diagnostic criteria for Mild Cognitive Impairment or Dementia 4. Majority of group has mild cognitive symptoms as evidenced by: a. MMSE ≥ 20 b. CDR < 2 c. Equivalent Measure					
Concept	1. Sleep measurement / assessment defined within aims and objectives of the study. 2. Validated sleep outcome measure / tool					
Context	Community and health-care settings					
Types of Evidence Source	1. Written in English Language 2. Peer Reviewed Published Articles 3. Interventional, observational or qualitative studies					
Details and characteristics of evidence source					
Date						
Author(s)						
Title						
Country						
Context	Community		Healthcare	
Participant Number						
Participant Gender (Female)						
Participant MMSE / CDR / Other						
Participant Diagnosis (%)	Mild Cognitive Impairment		Dementia	
Type of Dementia(s) (%)						
Type Of Study						
Interventional / Observational	Interventional		Observational	
Details/results extracted from evidence source					
Validated Outcome Measures						
Sleep Parameters Reported						

### Data analysis and presentation

Attributes of each included study will be listed (headings described in Table 2). 

**Table 2.** Individual study characteristics.

Table Heading	Description
Author / Year	Author and year study published
Interventional / Observational Study	Interventional or Observational Design
Study Design	Type of Study e.g. RCT / Cohort / Case-Control
Study Population	Number of Participants
Population Diagnosis	Diagnosis of Participants e.g. MCI / Early AD
Outcome Measure(s) Reported	Sleep Parameter(s) reported
Sleep Measurement Tool(s) Utilised	Validated sleep tool(s) utilised

**Figure 1.**
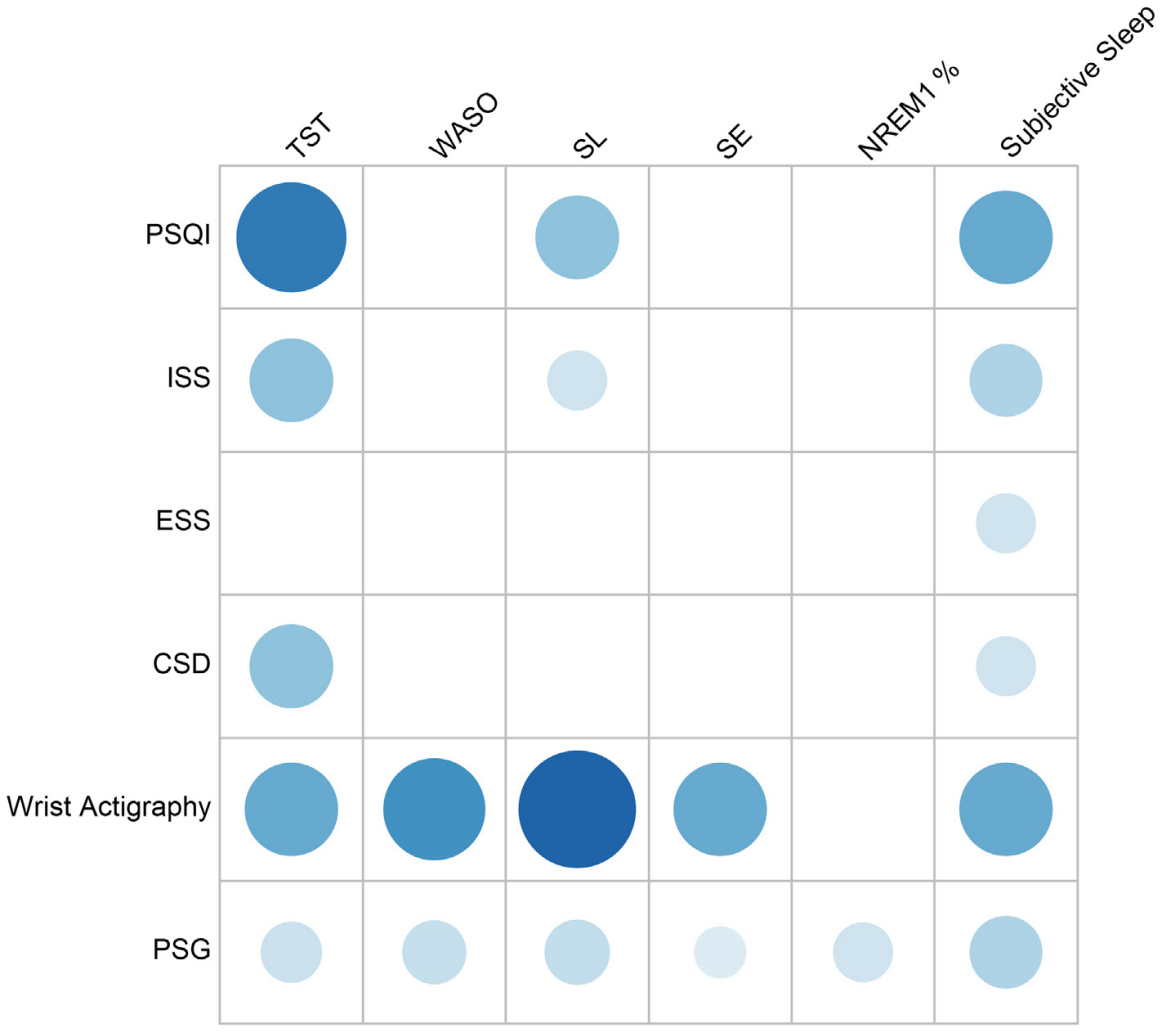
Balloon plot of frequency of reported parameters by sleep measure (example).

**Figure 2.**
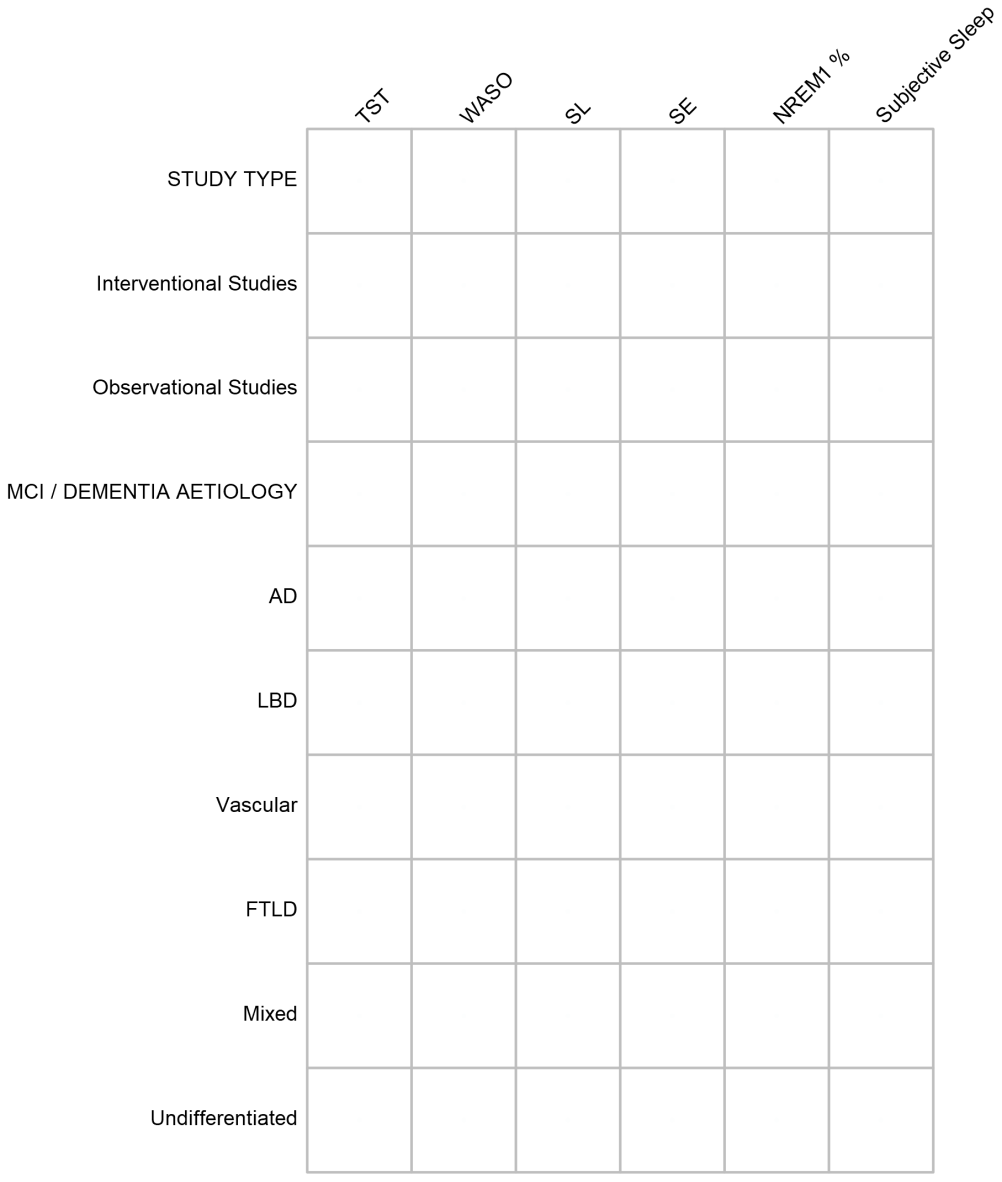
Stratified balloon plot template – sleep parameters.

**Figure 3.**
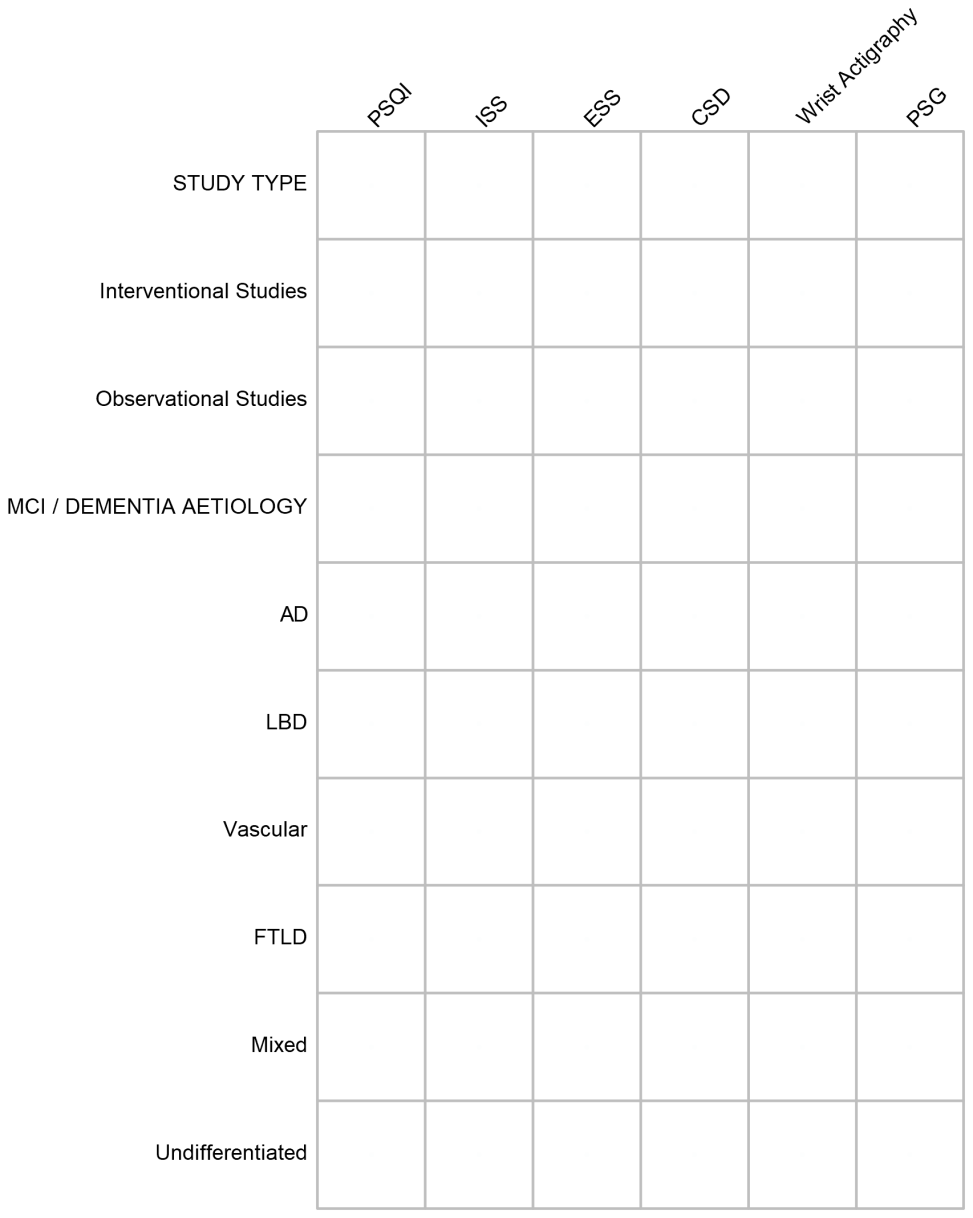
Stratified balloon plot template – measurement tools.

A balloon plot will be produced combining sleep outcome parameters on one axis with validated sleep tool on the other axis (see Figure 1 for example). Two further balloon plots will be produced. Each will stratify the group by participant diagnosis and also by study type. These will plot the proportion of studies reporting each sleep parameter (see Figure 2) and the proportion of studies utilising each sleep measurement tool (see Figure 3). This will be followed by a narrative summary of collected data. 

The results of this review will provide an understanding as to ways and means by which sleep is measured in this specific group allowing for identification of the tools and parameters most likely to facilitate comparison across studies. 

## Study status

Full search is currently pending.

## Dissemination

Results will be published in a peer-reviewed journal and presented at relevant academic conferences. The search strategy will be made available publicly for transparency. 

## Data availability

No data are associated with this article
