# Supplementary material for: Protocol for a scoping review of measurement of sleep in mild cognitive impairment and early dementia
Source: AMRC Open Res. 2021 Apr 14;3:13. [Version 1] doi: 10.12688/amrcopenres.12981.1 (PMC11064978; doi:10.12688/amrcopenres.12981.1)
Supplement: Peer review history

# Reviewer report: Reviewer response for version 1

**DOI:** 10.21956/amrcopenres.14054.r26761

**Published:** 2021-08-17

**Authors:** Mariana D’Amico

**Affiliations:** 1 Occupational Therapy, Tampa Bay Regional Campus, Nova Southeastern University, Tampa, FL, USA

This protocol for conducting a scoping review about sleep measurements used for individuals with mild cognitive impairment and early dementia is well written and thought out in terms of methods and parameters. Sleep is an essential health factor and identification of accurate measure is increasingly important. Completion of this protocol will add to the body of knowledge related to gaps in assessment and provide directions for future research.

Is the study design appropriate for the research question?

Yes

Is the rationale for, and objectives of, the study clearly described?

Yes

Are sufficient details of the methods provided to allow replication by others?

Yes

Are the datasets clearly presented in a useable and accessible format?

Yes

Reviewer Expertise:

mental health, elderly, children and youth, occupational engagement and assessments

I confirm that I have read this submission and believe that I have an appropriate level of expertise to confirm that it is of an acceptable scientific standard.

# Reviewer report: Reviewer response for version 1

**DOI:** 10.21956/amrcopenres.14054.r26669

**Published:** 2021-05-11

**Authors:** Andrea Tales

**Affiliations:** 1 Centre for Innovative Ageing, Swansea University, Swansea, UK

The article very clearly describes the protocol to determine the variety of sleep parameters currently reported and how they are measured in individuals living with mild cognitive impairment and the earlier stages of dementia.

 In my opinion the study design is appropriate for the research question. A potential area for further discussion is the rationale/decision to include the 'majority' of the study group with mild/early dementia /MCI ....why only the majority (i.e, equal or greater than 50%) rather than the whole of the study group having mild/early dementia/MCI? 

 What about potential bias in lack of publication of non-significant research findings?

Is the study design appropriate for the research question?

Yes

Is the rationale for, and objectives of, the study clearly described?

Yes

Are sufficient details of the methods provided to allow replication by others?

Yes

Are the datasets clearly presented in a useable and accessible format?

Yes

Reviewer Expertise:

Ageing and dementia research: EEG, psychophysics, neurocognition, vision & attention

I confirm that I have read this submission and believe that I have an appropriate level of expertise to confirm that it is of an acceptable scientific standard.
